# Postbiotic *Enterococcus faecalis* EF-2001 induces IL-10 production through macrophage scavenger receptor 1-mediated phagocytosis

**DOI:** 10.3389/fimmu.2026.1901007

**Published:** 2026-08-20

**Authors:** Yuki Kubota, Takumi Tsuboya, Marina Murakami, Shuichiro Uehara, Shigeo Suzuki

**Affiliations:** 1 Department of Pediatric Surgery, Nihon University School of Medicine, Tokyo, Japan; 2 Nihon Berumu Co., Ltd., Tokyo, Japan

**Keywords:** anti-inflammatory cytokine, *Enterococcus faecalis* EF-2001, immune modulation, lactic acid bacteria, macrophage scavenger receptor 1, phagocytosis, postbiotics, signaling pathway

## Abstract

Postbiotics have attracted attention owing to their safety and immunomodulatory effects. The heat-inactivated lactic acid bacterium *Enterococcus faecalis* EF-2001 (EF-2001) is one such postbiotic. Extensive studies on EF-2001 have demonstrated its safety and versatile biological activities, particularly immunomodulatory effects such as anti-inflammatory responses. However, the molecular mechanisms underlying these regulatory processes remain unclear. Here, we investigated phagocytosis and cytokine production of EF-2001 using murine macrophage-like cells, RAW264 cells. After stimulation with EF-2001, phagocytosis was evaluated using flow cytometry and microscopy. Secreted cytokines were evaluated using ELISA. RNA-seq and gene set enrichment analyses were performed to identify candidate receptors. To validate the candidate receptors, the receptor genes were downregulated by siRNA knockdown, followed by Quantitative PCR and immunoblotting to determine knockdown efficacy. The effects of gene silencing on cell function were characterized by flow cytometry and ELISA. A neutralizing antibody was used to confirm knockdown experiments. The intracellular signaling pathways were examined using specific inhibitors of key kinases. EF-2001 was phagocytosed by RAW264 cells with cytokine production, including IL-10, TNF-α, and IL-6, in a dose-dependent manner. Bioinformatic analysis identified *Msr1*, *Cd36* and *Colec12* as candidate receptor genes for phagocytosis by EF-2001. Phagocytosis was significantly reduced by suppressing macrophage scavenger receptor 1 (MSR1) expression. The neutralizing antibody against MSR1 showed similar suppression of phagocytosis. Thus, MSR1 was identified as the primary receptor for EF-2001 recognition and phagocytosis. MSR1 suppression also affects cytokine production, especially that of IL-10. In terms of intracellular signaling pathways, Syk and PI3K inhibition substantially reduced EF-2001 phagocytosis. The inhibition of Syk, PI3K, and MAPKs hampered IL-10 production induced by EF-2001 stimulation. EF-2001 is recognized and phagocytosed primarily via MSR1 in macrophages, accompanied by the induction of IL-10. Syk and PI3K signaling are involved in phagocytosis and IL-10 release, whereas their downstream MAPK pathways predominantly contribute to IL-10 production. Our data provide insights into the immunomodulatory mechanisms through which biotic components interact with macrophages.

## 1 Introduction

In the small intestine, antigens are taken up by microfold (M) cells, specialized epithelial cells located within the follicle-associated epithelium of Peyer’s patches and other lymphoid tissues. Antigens are delivered to the underlying immune tissues, initiating mucosal immune responses (1, 2). These antigens are recognized by phagocytes, such as macrophages, which contribute to the induction of adaptive immunity via antigen presentation (3, 4) and immune response regulation through both pro- and anti-inflammatory cytokine production (5, 6). Among the anti-inflammatory cytokines, interleukin-10 (IL-10) plays a pivotal role in regulating inflammatory responses by suppressing the production of pro-inflammatory cytokines, including TNF-α, and inhibiting the activation of the NF-κB signaling pathway (7). Dysregulation of macrophage function has been implicated in the pathogenesis of chronic inflammatory bowel diseases, including Crohn’s disease (8). These findings suggest that macrophages contribute to the maintenance of immune homeostasis by balancing pro- and anti-inflammatory signals.

Recognition of exogenous factors by macrophages is mediated by multiple receptors. These include pattern recognition receptors (PRRs) such as Toll-like receptors (TLRs), C-type lectin receptors (CLRs), and scavenger receptors (SRs) (9–11). SRs are involved in the recognition and uptake of various microorganisms, including pathogenic bacteria (11). They also interact with pathogen-associated molecular patterns (PAMPs), for example, lipopolysaccharide (LPS) and lipoteichoic acid (LTA) (11, 12). SRs are classified into 12 classes based on their gene sequences and protein structures. Class A and B receptors are involved in phagocytosis by gram-positive bacteria such as *Staphylococcus aureus* and *Listeria monocytogenes* (11, 12). Of these, macrophage scavenger receptor 1 (MSR1, also known as SR-A1 or CD204) and collecting subfamily member 12 (COLEC12, also known as SR-A4) belong to class A while CD36 belongs to class B. Phagocytosis mediated by MSR1 activates the mitogen-activated protein kinases (MAPKs) pathways, leading to the production of cytokines such as TNF-α (13). MSR1 may be involved in macrophage responses to Gram-positive bacteria, where it contributes to the regulation of the magnitude and quality of cytokine production (14). These findings suggest that SR-mediated phagocytosis, cytokine secretion, and signal transduction are important molecular mechanisms involved in the inflammatory responses of macrophages.

Probiotics are live microorganisms that are beneficial to the health of the host (15, 16). They modulate host immune responses, contributing to protection against infection and regulating inflammation (17). Lactic acid bacteria (LAB) are widely used probiotics. Numerous studies have demonstrated their beneficial effects, including prevention of various diseases, suppression of disease severity (18, 19), and modulation of immune function (17) in humans and animal models. Probiotics, which consist of live microorganisms, are commonly used in clinical practice. However, several probiotics are associated with infectious complications and other adverse events (20–22). This has raised concerns, particularly in the context of immunocompromised patients. These findings suggest that the appropriate use of probiotics should be carefully considered. In contrast, heat-inactivated preparations of LAB, that is, non-viable bacterial cells, offer safety advantages over live bacterial preparations (23). Non-viable bacterial cells have various beneficial effects, including immunomodulatory activities (23, 24). These heat-inactivated microbes or their components are defined as postbiotics according to the International Scientific Association of Probiotics and Prebiotics (ISAPP) (25). Strategies that use postbiotics to prevent disease through immune modulation have attracted the attention of scientists in recent years.

Postbiotic EF-2001 was produced by heat sterilization of *Enterococcus faecalis* strain EF-2001. It has been used as a biological response modifier (BRM), which has immunomodulatory effects (26). Extensive studies on EF-2001 have revealed a wide range of biological functions. In mouse models, the ingestion of EF-2001 ameliorated atopic dermatitis (27) and ulcerative colitis, along with the associated depressive and anxiety-like symptoms (28, 29). These studies demonstrated the suppression of inflammation by EF-2001. In humans, EF-2001 improves oral symptoms, in patients with oral candidiasis (30) and increases IgM secretion in healthy individuals (31). The safety of EF-2001 was confirmed in a subchronic oral toxicity study in mice (32). Whole-genome sequence analysis showed that it lacks major virulence factors (33). Therefore, these findings suggest that EF-2001 is a promising postbiotic because of its diverse biological functions and safety. These effects are mediated through actions on immune cells. However, the underlying molecular mechanisms remain unclear.

In this study, we aimed to elucidate the underlying mechanisms, how EF-2001 exerts the versatile effects, especially its anti-inflammatory effects, in animals and humans. We hypothesized that the immunomodulatory activity of EF-2001 was initiated by phagocytosis. Hence, phagocytosis and cytokine induction by EF-2001 were investigated, and its corresponding receptor was explored in murine macrophage model cells. The intracellular signaling pathways involved in these phenomena were analyzed. These results revealed the phagocytic receptor of EF-2001 and elucidated its activation mechanisms.

## 2 Materials & methods

### 2.1 Antibodies and inhibitors

Goat polyclonal anti-CL-P1/COLEC12 (cat. no. AF3130-SP), goat polyclonal anti-CD36 (cat. no. AF2519-SP), and goat polyclonal anti-SR-A1/MSR1 (cat. no. AF1797) antibodies were purchased from R&D Systems (Minneapolis, MN, USA). Rabbit polyclonal anti-GAPDH antibody (cat. no. 10494-1-AP), rabbit polyclonal anti-goat IgG (H + L)-HRP (cat. no. SA00001-4) and goat polyclonal anti-rabbit IgG (H+L)-HRP (cat. no. SA00001-2) were obtained from the Proteintech Group (Rosemont, IL, USA). Rat monoclonal anti-Mouse CD204 (clone 2F8, cat. no. MCA1322T) and rat IgG2b monoclonal isotype control (cat. no. MCA6006GA) antibodies were obtained from Bio-Rad Laboratories (Hercules, CA, USA). VX-745 (cat. no. SML1638-5MG) was purchased from Sigma-Aldrich (St. Louis, MO, USA). Wortmannin (cat. no. S2758), and piceatannol (cat. no. S3026), TJ-M2010-5 (cat. no. E1721), PD184352 (cat. no. S1020), and JNK-IN-8 (cat. no. S4901) were purchased from Selleck Chemicals (Houston, TX, USA).

### 2.2 Postbiotics and preparation

Commercially available EF-2001, originally isolated from healthy human feces, was obtained as a heat-killed dried powder from Nihon Berumu Co., Ltd. (Tokyo, Japan). The bacterial count concentration of EF-2001 is 7.5 ×10^12^ cells/g, and it contained no excipients or other additives. EF-2001 was suspended in PBS before use in all the experiments. For the phagocytosis assay, an EF-2001 suspension in PBS was ultrasonicated for 2 min. EF-2001 cells were labeled with 1 mg/mL FITC-I (Dojindo Laboratories, Kumamoto, Japan) for 1 h and washed thrice with PBS before use.

### 2.3 Cell culture

RAW264 cells were obtained from the RIKEN Bioresource Center (Ibaraki, Japan). The cells were cultured in RPMI-1640 (FUJIFILM Wako Pure Chemical Corporation, Osaka, Japan) supplemented with 10% fetal bovine serum (FBS) (BioSera, Cholet, France), 100 U/mL penicillin–streptomycin (FUJIFILM Wako Pure Chemical Corporation), and 10 mM HEPES (Nacalai Tesque, Kyoto, Japan). The cells were incubated at 37 °C in a humidified atmosphere containing 5% CO_2_.

### 2.4 RNAi knockdown

The siRNAs for *Msr1*, *Colec12*, and *Cd36* were designed and synthesized by Nippon Gene (Tokyo, Japan), and a universal negative control was purchased from Nippon Gene. The sequences were as follows: *Msr1*-siRNA passenger strand 5’-GAGGUAUUCCAGGUGUUAA-3,’ guide strand 5’-UUAACACCUGGAAUACCUC-3’; *Colec12*-siRNA passenger strand 5’-AGACUGUGCUGGCUUGAUU-3,’ guide strand 5’-AAUCAAGCCAGCACAGUCU-3’; *Cd36*-siRNA passenger strand 5’-AAGCCGAUCUACAAAAUGA-3,’ guide strand 5’-UCAUUUUGUAGAUCGGCUU-3.’ siRNA complexes were prepared using Opti-MEM I Reduced Serum Medium (Thermo Fisher Scientific, Waltham, MA, USA) mixed with the TransIT-X2 Delivery System (Takara Bio, Shiga, Japan) and added to pre-cultured cells at a final concentration of 50 nM, followed by incubation for 24 h.

### 2.5 Receptor blockade

Cells were pre-treated with a neutralizing (anti-CD204, Bio-Rad) or isotype control (rat IgG2b, Bio-Rad) antibody at a final concentration of 1 µg/mL for 1 h before the phagocytosis assay.

### 2.6 Inhibition of signal transduction pathways

The cells were pre-treated for 1 h with signal transduction inhibitors (20 µM piceatannol, 20 µM TJ-M2010-5, 0.5 µM wortmannin, 10 µM JNK-IN-8, 10 µM PD184352, and 0.5 µM VX-745 against Syk, MyD88, PI3K, JNK, ERK1/2, and p38, respectively) dissolved in DMSO. PD184352 was used as an ERK1/2 inhibitor because it selectively inhibits MEK1/2, which is upstream of ERK1/2, blocking ERK1/2 activation. The concentration of inhibitors were determined based on the previous study (34). Cytokine production and phagocytosis assays were performed under the conditions described in each respective assay section.

### 2.7 RNA isolation and quantitative PCR

For gene expression analysis, RAW264 cells were seeded into 24-well plates at a density of 1.5 × 10^5^ cells/well and incubated for 16–24 h. The cells were then transfected with siRNAs for 24 h as described in the RNAi Knockdown section. Total RNA was extracted from the knockdown cells using the ReliaPrep™ RNA Miniprep System (Promega Corporation, Madison, WI, USA). cDNA was synthesized using the PrimeScript RT Reagent Kit (Takara Bio) according to the manufacturers’ instructions. Quantitative PCR (qPCR) was performed using TB Green Premix Ex Taq™ II FAST qPCR (Takara Bio) with an intercalating dye-based method, using the synthesized cDNA as a template. Thermal cycling conditions consisted of an initial denaturation at 95 °C, followed by 40 cycles of denaturation at 95 °C, and annealing/extension at 60 °C. qPCR amplification and fluorescence detection were performed using qTOWER³ real-time PCR system (Analytik Jena AG, Jena, Germany). The primers used for qPCR were obtained from FASMAC (Kanagawa, Japan). Relative gene expression levels were normalized to *Tbp*, an endogenous control gene, and calculated using the standard curve method. Amplification specificity was confirmed using melting curve analysis. The sequences of primers used in this study were as follows: *Tbp*, Forward 5’-ACCGTGAATCTTGGCTGTAAAC-3,’ Reverse 5’-GCAGCAAATCGCTTGGGATTA-3’; *Msr1*, Forward 5’-GAGTCCGTGAATCTACAGCAA-3,’ Reverse 5’-AGTCTGAGGTCGTTGGTGAT-3’; *Colec12*, Forward 5’-TGGATCGGCCTCACAGACTC-3,’ Reverse 5’-AGTCATTCCACTGTCCTGCGTA-3’; *Cd36*, Forward 5’-GATGACGTGGCAAAGAACAG-3,’ Reverse 5’-CAGTGAAGGCTCAAAGATGG-3.’

### 2.8 Protein expression analysis

RAW264 cells were seeded into 6-well plates at a density of 6.5 × 10^5^ cells/well and incubated for 16–24 h. To evaluate the effect of gene silencing at the protein level, cells were transfected with siRNA for 24 h as described in the RNAi Knockdown section. Following incubation, the cells were lysed in RIPA buffer (FUJIFILM Wako Pure Chemical Corporation) supplemented with cOmplete™ Mini Protease Inhibitor Cocktail (Sigma-Aldrich). Genomic DNA was sheared by sonication and the lysates were centrifuged (10,000×*g*, 10 min) using the High Speed Microcentrifuge Micro-12 (Greiner Bio-One, Kremsmünster, Austria). The supernatants were collected and protein concentrations were measured using a BCA protein assay kit (Nacalai Tesque). Equal amounts of protein were loaded onto 5%–20% gradient polyacrylamide gels and electrophoresed. This was followed by transfer onto PVDF membranes (Bio-Rad) using the Trans-Blot Turbo System (Bio-Rad). Blocking and antibody incubations were performed using the iBind™ Flex Western System (Thermo Fisher Scientific) according to the manufacturer’s instructions. Primary antibodies were used at a dilution of 1:200 for goat anti-CL-P1/COLEC12 and goat anti-CD36, 1:2000 for goat anti-SR-A1/MSR1, and 1:5000 for rabbit anti-GAPDH. Secondary antibodies were used at a dilution of 1:1000, except for SR-A1/MSR1, for which a 1:500 dilution was used. The protein–antibody complexes were visualized using Pierce™ ECL Plus Western Blotting Substrate (Thermo Fisher Scientific). The immunoreactive protein bands were detected using a C-DiGit™ Chemiluminescence Scanner (LI-COR, Lincoln, NE, USA).

### 2.9 Cytokine production assay

RAW264 cells were seeded into 96-well plates at a density of 5.0 × 10^4^ cells/well and incubated for 16–24 h. To determine the dose response, cells were stimulated with EF-2001 at a final concentration of 25, 50, and 100 µg/mL for 6 or 24 h. The cytokine measurement time points were selected based on unpublished preliminary optimization experiments. For siRNA or inhibitor experiments, the cells were seeded at 3.7 × 10^4^ cells/well and incubated for 16–24 h. After the respective pre-treatments (siRNA for 24 h, or inhibitors for 1 h as described in the RNAi Knockdown, Receptor Blockade, and Inhibition of Signal Transduction Pathways sections), the cells were stimulated with 50 µg/mL EF-2001 for 6 h. Cells were pre-treated with 0.25 or 2 µM cytochalasin D (CytD) for 1 h before EF-2001 stimulation to inhibit phagocytosis. Following stimulation, the culture supernatants were collected. The levels of mouse IL-6, IL-10, and TNF-α were quantified using Mouse IL-10 DuoSet ELISA, Mouse TNF-α DuoSet ELISA, and Mouse IL-6 DuoSet ELISA (R&D Systems) according to the manufacturer’s instructions. TMB Solution (FUJIFILM Wako Pure Chemical Corporation) and 1 M sulfuric acid (Nacalai Tesque) were used for color development and reaction termination, respectively. The absorbance (450 nm) was measured using a Varioskan LUX Multimode Microplate Reader (Thermo Fisher Scientific).

### 2.10 Phagocytosis assay

The EF-2001 uptake assay was performed as previously described with minor modifications (35). In brief, RAW264 cells were seeded into 12-well plates at a density of 3.0 × 10^5^ cells/well. To maintain consistency across experiments, the cells were incubated for either 24 h for basic uptake and siRNA experiments, or 48 h for neutralizing antibody and inhibitor experiments to match the respective pretreatment protocols. As a positive control for phagocytosis inhibition, the cells were pre-treated with 0.25 µM CytD for 1 h. FITC-labeled EF-2001 was added to the cells at a final concentration of 50 µg/mL and incubated for 2 h. After quenching extracellular fluorescence with Trypan Blue (Nacalai Tesque), the cells were fixed with 4% paraformaldehyde (Nacalai Tesque) and analyzed using the CytoFLEX System B3-R3-V0 (2L6C) (Beckman Coulter, Brea, CA, USA). Data analysis was performed using the CytExpert software (version 2.6, Beckman Coulter). Phagocytic activity was quantified as the phagocytosis index, which was represented by the product of the number of FITC-positive cells and the mean fluorescence intensity (MFI).

### 2.11 Cell viability assay

To assess the effects of CytD and signal transduction inhibitors on cell viability, RAW264 cells were seeded into 96-well plates at a density of 5.0 × 10^4^ cells/well and incubated for 24 h. The cells were then treated with 0.25 or 2 µM CytD, or with the signal transduction inhibitors at the concentrations described in the Inhibition of Signal Transduction Pathways section, for 1 h. Cell viability was assessed using a Cell Counting Kit-8 (CCK-8; FUJIFILM Wako Pure Chemical Corporation) according to the manufacturer’s instructions. After treatment, CCK-8 reagent was added to each well, and the cells were incubated for 1 h at 37 °C in a humidified atmosphere containing 5% CO_2_. Absorbance was measured at 450 nm using a Varioskan LUX Multimode Microplate Reader (Thermo Fisher Scientific). Cell viability was calculated relative to the corresponding control group, which was set to 100%.

### 2.12 Microscopy

RAW264 cells were seeded at a density of 5.0 × 10^4^ cells/well onto glass coverslips in 24-well plates and cultured for 16–24 h. To evaluate phagocytosis, cells were pre-treated for 1 h with or without CytD (FUJIFILM Wako Pure Chemical Corporation) in DMSO, followed by incubation with FITC-labeled EF-2001 (final concentration: 200 µg/mL) for 2 h at 37 °C. After incubation, the extracellular fluorescence was quenched by treating the cells with Trypan Blue (Nacalai Tesque). The cells were then fixed with 100% ethanol at −20 °C. Nuclei were stained with DAPI (Nacalai Tesque, Kyoto, Japan). Glass coverslips were mounted on glass slides and sealed with the VECTASHIELD Vibrance Antifade Mounting Medium (Vector Laboratories, Burlingame, CA, USA). Images were acquired using a fluorescence microscope (BX53, Olympus, Tokyo, Japan) equipped with a 40× objective lens and digital color camera (DP22, Olympus). Bright-field images were acquired under the same conditions, using the same microscope and camera system. Fluorescence images were captured with fixed settings: an exposure time of 1/50 s and a constant excitation intensity (level 12 on a 0%–100% scale) for both channels. Images were analyzed and merged using ImageJ software (version 1.54g) (36).

### 2.13 RNA-seq and bioinformatics analyses

RAW264 cells were stimulated with 100 µg/mL EF-2001 for 6 h. Total RNA was extracted from the cells before and after stimulation using the ReliaPrep™ RNA Miniprep System (Promega Corporation) according to the manufacturer’s instructions. RNA sequencing analyses were performed by Takara Bio, and the sequencing results were provided in the FastQ file format. The data have been deposited with links to BioProject accession number PRJDB42382 in the DDBJ BioProject database. Raw sequencing reads were pseudo-aligned to the mouse genome (GRCm39, release 115) using Salmon software (version 1.10.3). Subsequent analyses were performed using R (version 4.5.0, 2025-04-11) in the RStudio environment (version 2026.01.1 + 403). Raw counts were filtered (count > 10) and normalized to transcripts per million (TPM) using the tximport (version 1.38.2) and DESeq2 (version 1.50.2) software. The normalized genes were ranked by the Wald statistic and subjected to pre-ranked gene set enrichment analysis (GSEA) for investigation of gene set enrichment using fgsea (version 1.36.2). Gene Ontology (GO) Biological Process gene set collection (m5.go.bp.v2026.1. Mm.symbols.gmt) was downloaded from the Molecular Signatures Database repository (MSigDB). The results were visualized using ggplot2 (version 4.0.2). Leading-edge genes detected in GO: BP_PHAGOCYTOSIS were combined with the genes in GO: MF_SCAVENGER_RECEPTOR_ACTIVITY downloaded from MSigDB. A Venn diagram of the combined data was plotted using Venn Diagram (version 1.8.2).

### 2.14 Statistics

Statistical analyses were performed using EZR (version 1.67; R version 4.5.1; Jichi Medical University, Tochigi, Japan) (37), which is a graphical user interface for R (The R Foundation for Statistical Computing, Vienna, Austria). Data are presented as the mean ± standard deviation (SD) of triplicate samples. Statistical comparisons between two groups were performed using the Student’s *t*-test. Differences among groups were evaluated using one-way analysis of variance (ANOVA) followed by Dunnett’s multiple comparison test for comparisons against the control group. *p* < 0.05 was considered statistically significant. Formal tests for normality and homogeneity of variance were not performed because of the small sample size (n=3). Parametric analyses were conducted under the assumptions of approximate normality and homogeneity of variance.

## 3 Results

### 3.1 EF-2001 is phagocytosed by RAW264 cells and induced pro- and anti-inflammatory cytokine production

**Figure 1:**
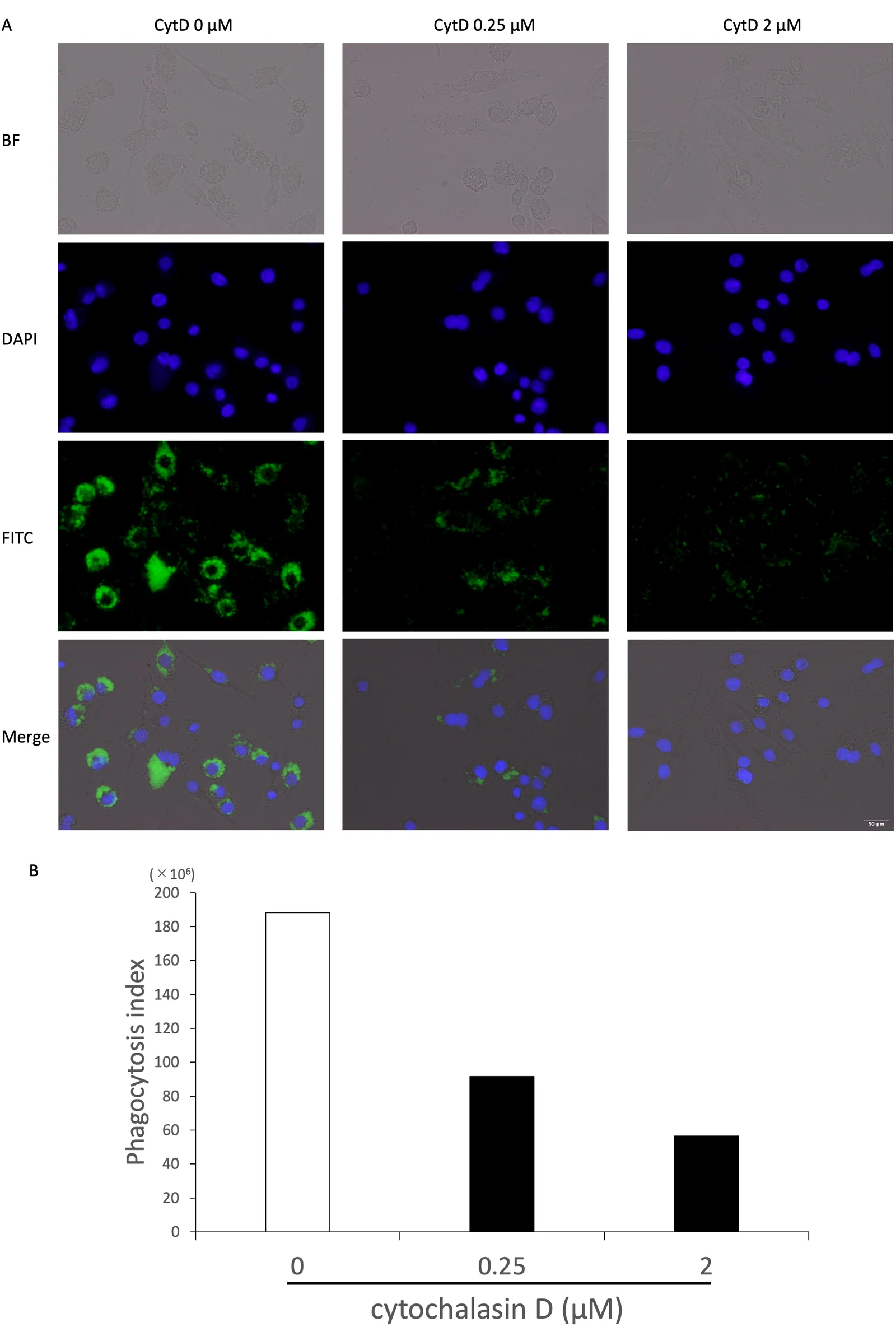
Phagocytosis of *Enterococcus faecalis* EF-2001 by RAW264 cells. **(A)** Representative fluorescence microscopy (FM) and bright-field (BF) images showing phagocytosis of FITC-labeled *Enterococcus faecalis* EF-2001 (EF-2001). Cells were pre-treated with or without cytochalasin D (CytD) for 1 h, followed by incubation with FITC-labeled EF-2001 (200 µg/mL) for 2 h. Nuclei were stained with DAPI (blue), and EF-2001 was labeled with FITC (green). Scale bar = 50 µm. Images were acquired using a 40× objective lens and a digital color camera. **(B)** Quantitative analysis of EF-2001 phagocytosis by flow cytometry (FCM). Cells were pretreated with the indicated concentrations of CytD for 1 h, followed by incubation with FITC-labeled EF-2001 (50 µg/mL) for 2 h. The phagocytosis index was calculated by multiplying the number of FITC-positive cells by the mean fluorescence intensity (MFI). For quenching extracellular fluorescence, the stained cells were treated with Trypan Blue before detection with FM **(A)** or FCM **(B)**. The data shown are representative results from multiple independent experiments.

**Figure 2:**
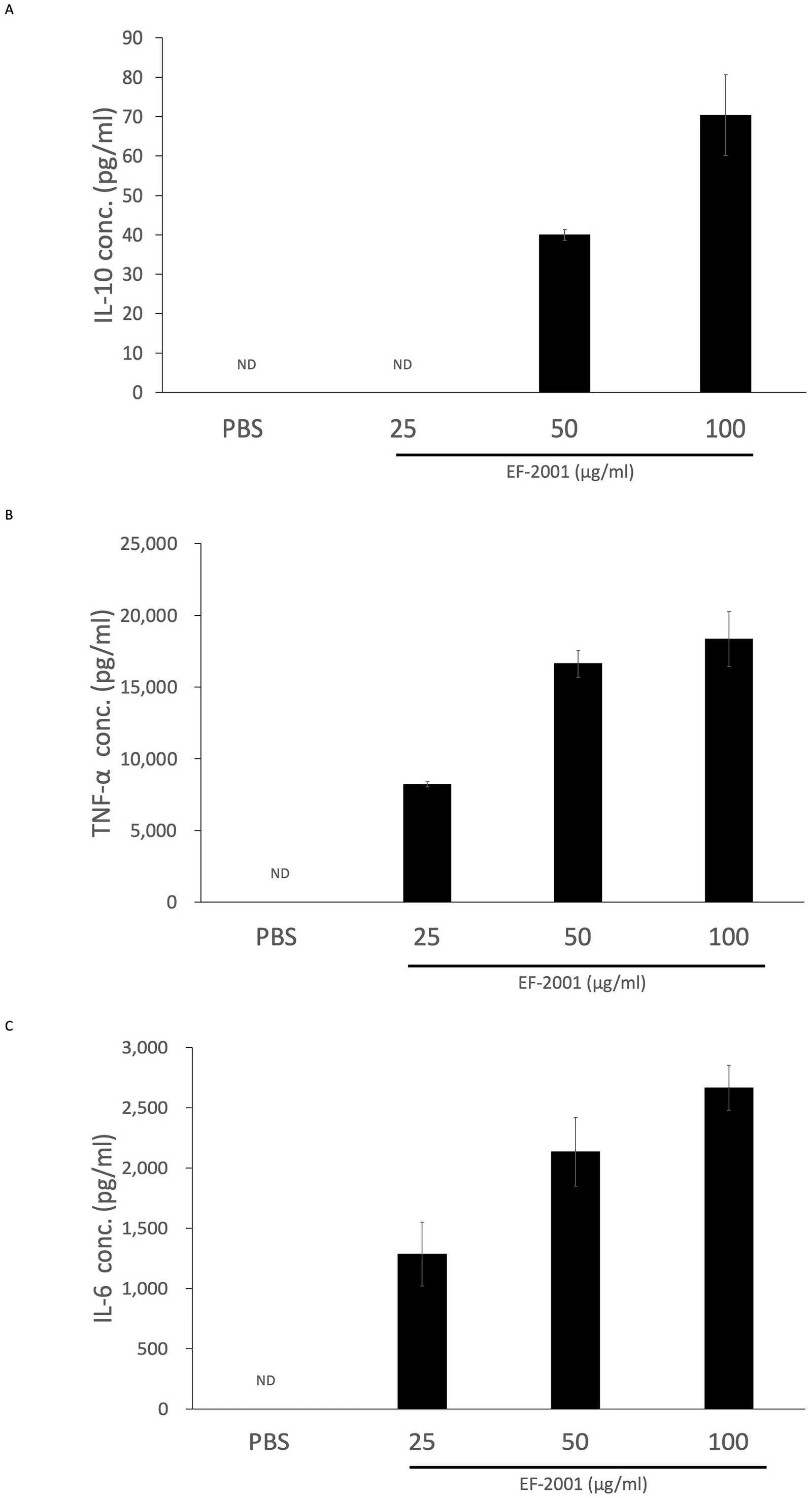
*Enterococcus faecalis* EF-2001 induces cytokine production by RAW264 cells. RAW264 cells were stimulated with *Enterococcus faecalis* EF-2001 at 25, 50, and 100 µg/mL for 6 h (**(A)** IL-10, **(B)** TNF-α) or 24 h (**(C)** IL-6). Cytokine levels in the culture supernatants were determined by ELISA. Data are presented as the mean ± SD of triplicate samples. ND, not detected.

To clarify the immune response to EF-2001, phagocytosis of EF-2001 was initially assessed to determine its capacity for cellular internalization. FITC-labeled EF-2001 was added to RAW264 cells, which are murine macrophage-like cells widely used for phagocytosis experiments. Fluorescence microscopy indicated that FITC-labeled EF-2001 was phagocytosed by the cells after 2 h of co-incubation (**Figure 1A**). This uptake was inhibited by CytD, an actin polymerization inhibitor. To quantify phagocytic activity, RAW264 cells treated with FITC-labeled EF-2001 were analyzed using flow cytometry (FCM) (**Figure 1B**). This also showed that the phagocytic activity was diminished in a concentration-dependent manner by CytD. Since the extracellular fluorescence was quenched with Trypan Blue, these data indicated the internalization of EF-2001 by phagocytosis. In addition to phagocytosis, the effect of EF-2001 on cytokine production was examined. RAW264 cells were treated with a series of concentrations of EF-2001 (25–100 µg/mL) and the supernatants were analyzed using ELISA. The stimulation led to dose-dependent increases in the production of IL-10 (**Figure 2A**) as well as TNF-α and IL-6 (**Figures 2B**, **C**).

### 3.2 RNA-seq in combination with gene enrichment analysis narrowed down expressed genes to candidate genes of phagocytic receptors of EF-2001

**Figure 3:**
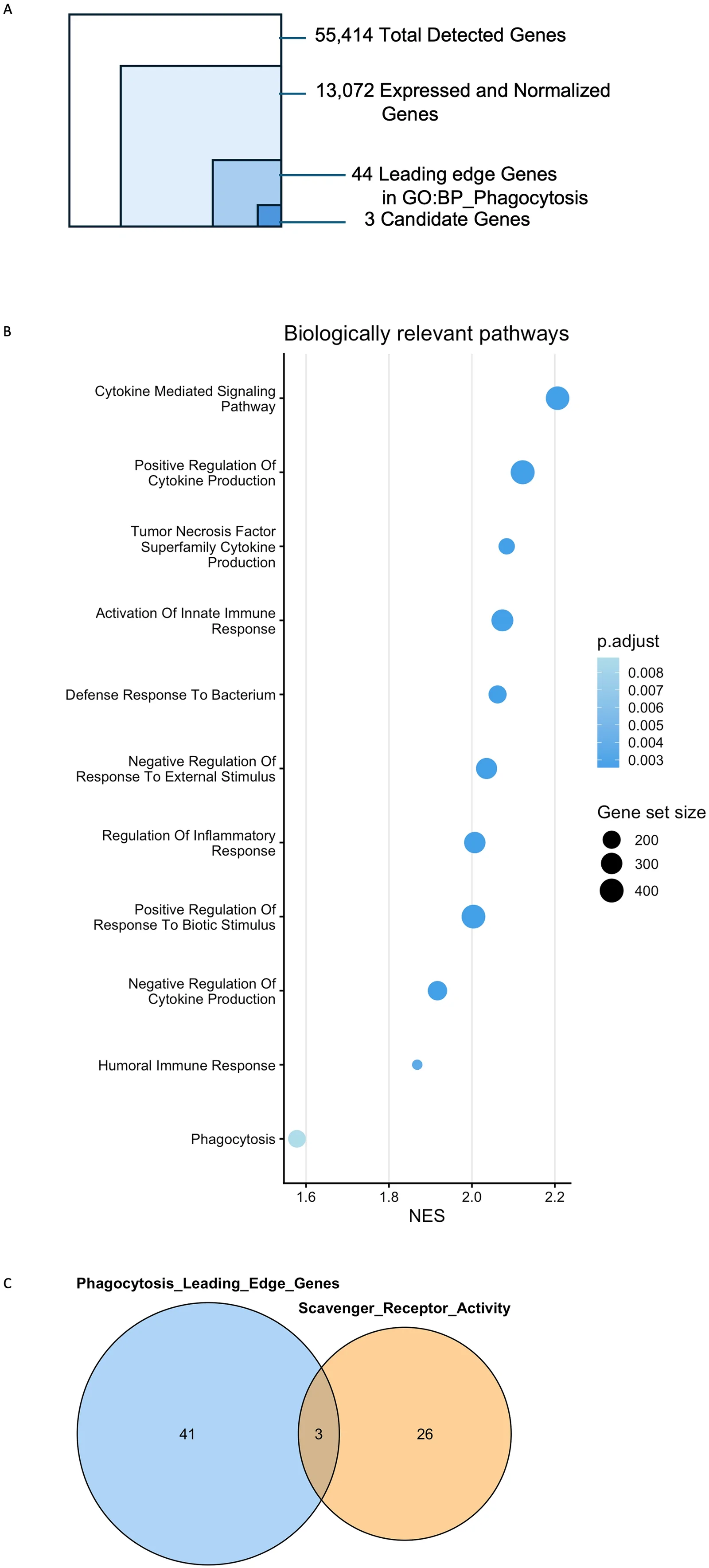
RNA-seq and Gene Set Enrichment Analyses narrow down candidate phagocytosis receptors for *Enterococcus faecalis* EF-2001. **(A)** Schematic representation of the candidate selection process. All transcripts (n=3) were filtered (gene counts > 10) and normalized using DESeq2. **(B)** The genes were ranked by the Wald statistic and analyzed by pre-ranked Gene Set Enrichment Analyses (GSEA). Leading-edge genes in GO: BP_PHAGOCYTOSIS were selected. Then, candidate genes were further narrowed down by combining the selected genes with genes in GO: MF_SCAVENGER_RECEPTOR_ACTIVITY. Gene ontology (GO) terms were selected based on relevance to the observed phenotype of EF-2001-stimulated RAW 264 cells. X-axis illustrate a normalized enrichment score (NES). The adjusted *p* value and gene set size are illustrated as dot color and size, respectively. **(C)** Venn diagram showing the intersection of two gene sets described above. Each gene set is listed in **Table 1**.

Next, we identified the receptor(s) related to the observed phagocytosis of EF-2001, since receptors are key molecules for recognition and phagocytosis. To identify candidate genes involved in EF-2001 phagocytosis, transcriptomic analysis and GSEA were performed to detect genes significantly enriched by EF-2001 stimulation. The selection process for candidate genes is summarized in **Figure 3A**. mRNA was extracted from RAW264 cells before and 6 h after stimulation with EF-2001 and reverse-transcribed into cDNA, followed by RNA-seq analysis. A total of 55,414 detected genes were filtered (count > 10) and normalized to identify 13,072 expressed genes. The genes were ranked using Wald statistic and analyzed using pre-ranked GSEA. GO terms with adjusted *p* values < 0.05 with Normalized Enrichment Score (NES) > 1.5 were selected as significant terms. To characterize the effects of EF-2001 on RAW264 cells, the relevant terms for the phenotypes shown in **Figure 1** and **2** were selected from the significant terms. The pathways associated with phagocytosis and cytokine production were selected (**Figure 3B**). The phagocytic pathway was significantly enriched (adjusted *p* value = 0.013) with NES = 1.582. Forty-four genes in the phagocytic pathway were identified as leading-edge genes, including several receptor genes (**Table 1**). Given that scavenger receptors play key roles in bacterial phagocytosis (11), we focused on the scavenger receptor activity pathway for corresponding molecular functions. The detected leading-edge genes were combined with the genes listed in GO: MF_SCAVENGER_RECEPTOR_ACTIVITY to further screen for receptor genes that recognize and engulf EF-2001 (**Figure 3C**). The genes used in the analysis are listed in **Table 1**. The combination of biological processes and molecular functions led to the identification of three common genes: *Msr1*, *Cd36*, and *Colec12*. In summary, the filtering strategy narrowed down to three candidate genes for the receptors for EF-2001 phagocytosis.

**Table 1:** List of gene sets used for finding common genes.

Gene set name	Gene symbol
Leading-edge genes in GO:BP_PHAGOCYTOSIS	Ccl2;Tgm2;Hck;Rab20;Syk;Hspa8;Anxa3;Jmjd6;Nos2;Slc11a1;Slc48a1;Fcgr2b;Msr1;Ldlr;Stap1;Ptprj;Trex1;Il2rg;Arap1;Tm9sf4;Tgfb1;Stat3;Colec12;Cd47;Pla2g5;Tlr2;Abr;Ncf4;Cd36;Myo1g;Nod2;Clcn3;Rapgef1;Cyba;Itgb2;Anxa1;Cln3;Vav1;Rab7;Pld4;Itgb1;Itgal;C3;Rab5a
Genes listed in GO:MF_SCAVENGER_RECEPTOR_ACTIVITY	Ssc4d;Cd36;Scarb2;Cd6;Ackr3;Dmbt1;Colec12;Enpp1;Enpp2;Endou;Lgals3bp;Stab1;Stab2;Msr1;Scarb1;Megf11;Vtn;Scarf2;Megf6;Scart1;Scart2;Ackr4;Ssc5d;Scarf1;Cxcl16;Megf10;Scara5;Cd163;Prg4

### 3.3 Knockdown effectively suppressed expression of candidate receptor genes and proteins

**Figure 4:**
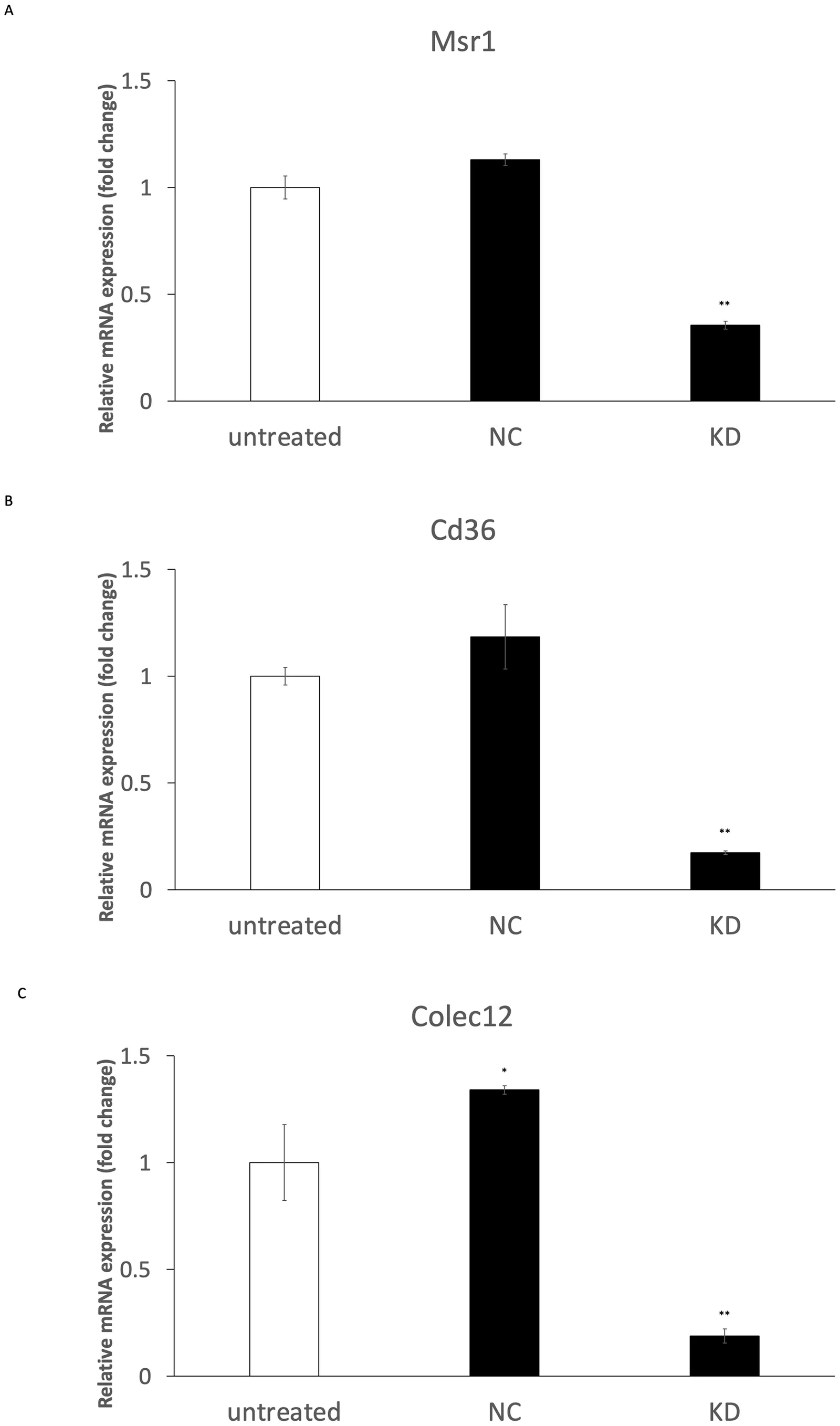
siRNA-mediated gene silencing effectively downregulates candidate phagocytosis receptors. RAW264 cells were transfected with 50 nM of siRNAs targeting each candidate gene for 24 h. Knockdown efficiency of mRNA expression of *Msr1* **(A)**, *Cd36* **(B)**, and *Colec12* **(C)** was confirmed by qPCR normalized to *Tbp*. Relative mRNA expression levels are presented as the mean ± SD (*n* = 3), with the expression in untreated cells set to 1.0. NC, universal negative control. Statistical significance was determined by one-way ANOVA followed by Dunnett’s multiple comparison test; *p < 0.05, **p < 0.01 versus the untreated group.

**Figure 5:**
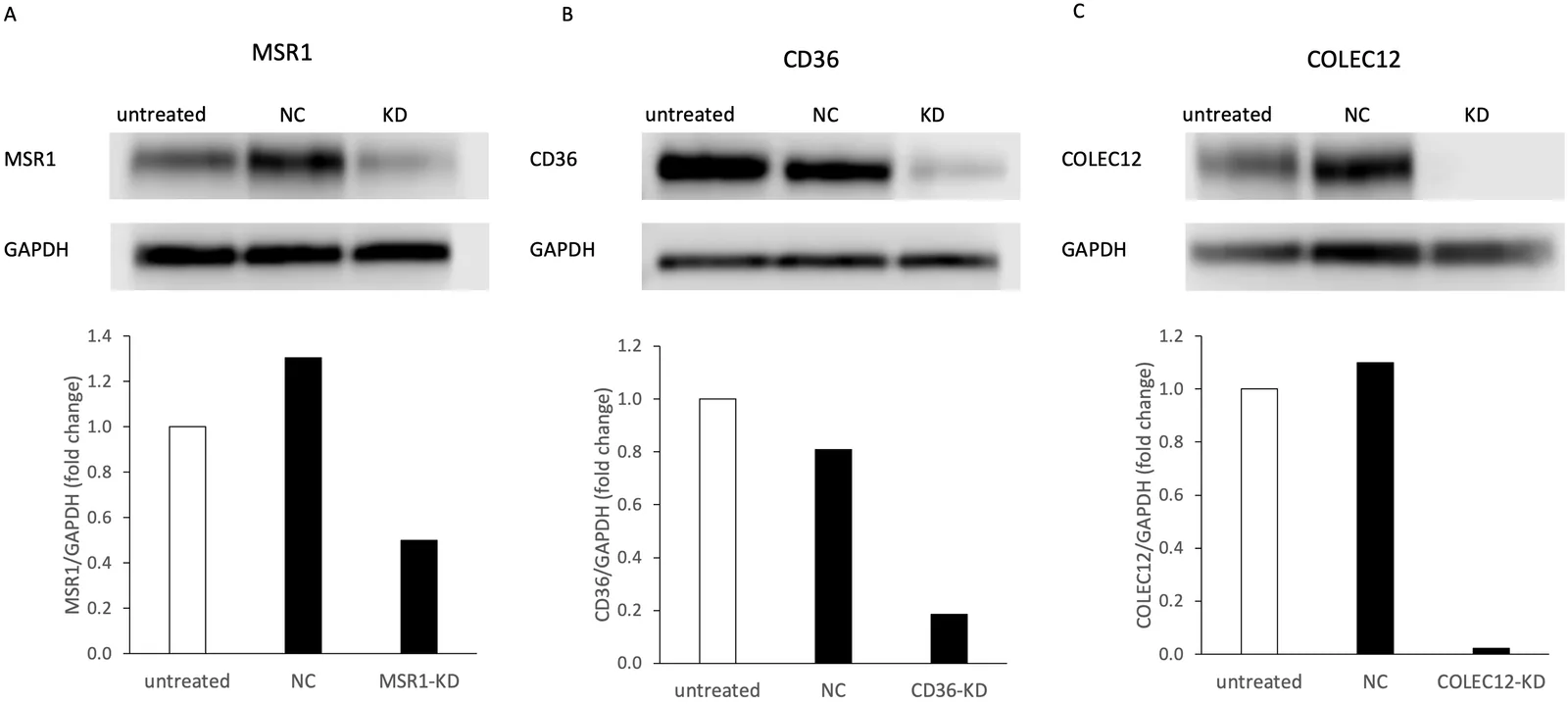
siRNA-mediated silencing effectively reduces protein expression of candidate receptors. RAW264 cells were lysed 24 h after siRNA transfection followed by measurement of protein expression levels of MSR1 **(A)**, CD36 **(B)** and COLEC12 **(C)** by immunoblotting. GAPDH was used as a loading control. NC was transfected with a universal negative control siRNA. Band intensities were quantified and normalized to GAPDH. Data are expressed as fold change relative to untreated cells. The data shown are representative results from multiple independent experiments.

To validate whether our selection process worked correctly, the functions of the candidate genes were confirmed using siRNA downregulation. The knockdown efficiency of siRNAs targeting the candidate receptor genes was evaluated. RAW264 cells were transfected with 50 nM of each siRNA (*Msr1*, *Colec12*, and *Cd36*) for 24 h. After analyte preparation, changes in the mRNA and protein expression levels of the target genes were evaluated by qPCR and immunoblotting, respectively. All the gene expression levels were significantly reduced compared with the untreated group by 64% for *Msr1*, 83% for *Cd36*, and 81% for *Colec12* (*p* < 0.01) (**Figure 4**). Similarly, all the protein expression levels were decreased compared with the untreated group by 51% for MSR1, 78% for CD36 and 91% for COLEC12 24 h after siRNA transfection (**Figure 5**). These RNAi data suggested that the knockdown efficiency was sufficient for subsequent functional analyses.

### 3.4 Gene silencing of candidate receptors dampened cytokine production in macrophages

**Figure 6:**
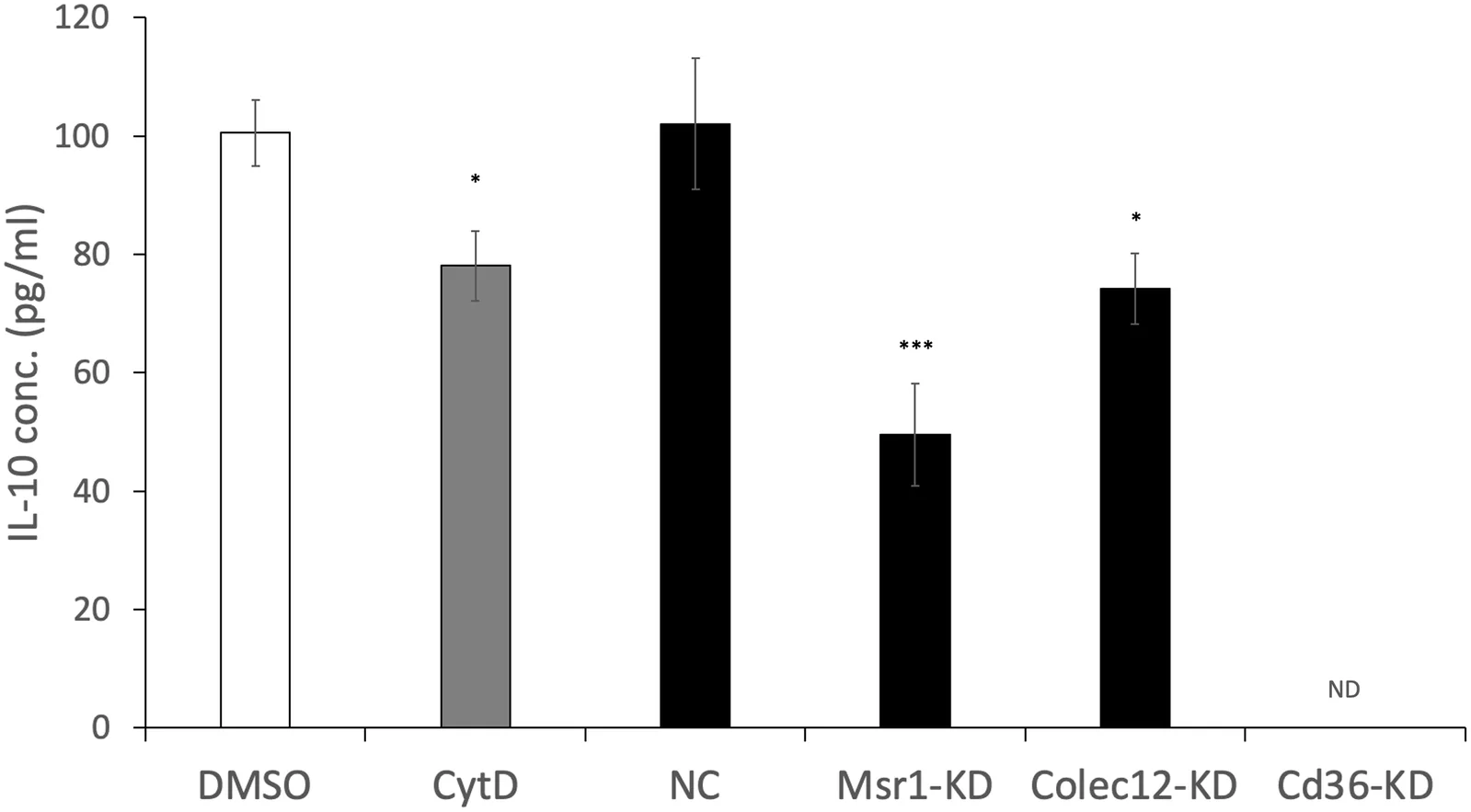
siRNA-mediated silencing of candidate receptors suppresses IL-10 production in response to *Enterococcus faecalis* EF-2001. RAW264 cells were transfected with siRNAs targeting each candidate gene for 24 h, followed by stimulation with *Enterococcus faecalis* EF-2001 (EF-2001) (50 µg/mL). IL-10 levels in the culture supernatant were determined by ELISA at 6 h post-stimulation. 0.25 µM cytochalasin D (CytD) was used as a positive control. Data are presented as the mean ± SD (n=3). NC, universal negative control; ND, not detected. Statistical significance was determined by one-way ANOVA followed by Dunnett’s multiple comparison test; *p < 0.05 ***p < 0.001 versus the NC group.

SR-mediated microbial phagocytosis in macrophages is associated with cytokine responses (38). Therefore, we hypothesized that the pro- and anti-inflammatory cytokines induced by EF-2001 stimulation were related to phagocytosis. RAW264 cells were transfected with siRNA for 24 h and stimulated with 50 µg/mL EF-2001 for 6 h to verify the contribution of each receptor to cytokine production. Compared to the negative control, downregulation of *Msr1* and *Colec12* significantly reduced the production of the anti-inflammatory cytokine IL-10, with moderate and slight effects, respectively (*p* < 0.001 and *p* < 0.01, respectively). Meanwhile, downregulation of *Cd36* drastically reduced production below the detection limit (**Figure 6**). In terms of pro-inflammatory cytokine, knockdown of *Cd36* significantly and modestly reduced TNF-α (*p* < 0.001) although those of *Msr1* and *Colec12* did not affect its production (**Supplementary Figure 1A**). IL-6 production significantly and moderately decreased (*p* < 0.001) after *Msr1*, *Cd36* and *Colec12* knockdown (**Supplementary Figure 1B**). The data showed a relationship between candidate receptors and cytokine release induced by EF-2001 stimulation.

### 3.5 MSR1 serves as the primary receptor for EF-2001 phagocytosis

**Figure 7:**
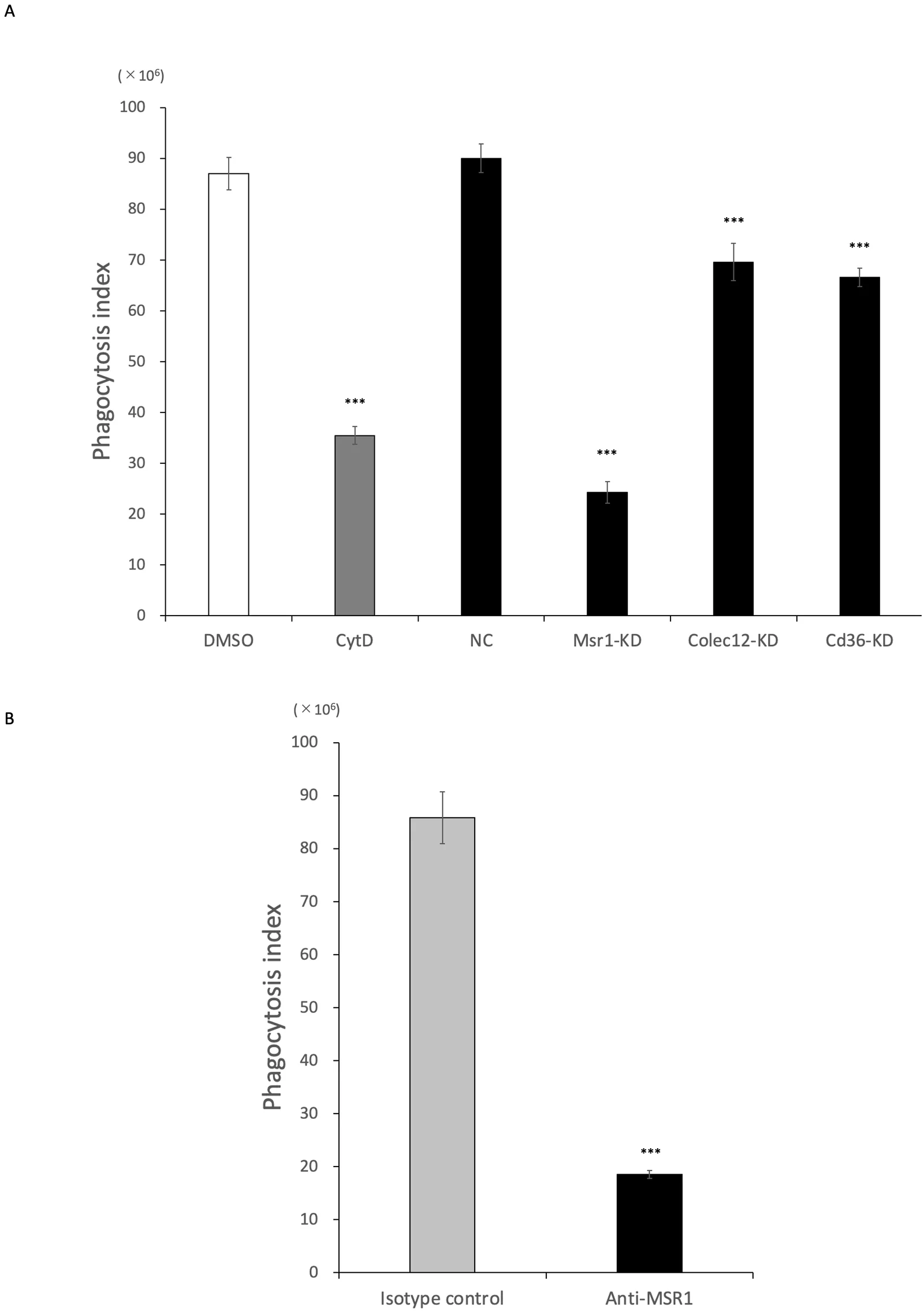
siRNA-mediated silencing and antibody blockade of candidate receptors suppress the phagocytosis of *Enterococcus faecalis* EF-2001. RAW264 cells were transfected with siRNAs targeting each candidate gene for 24 h **(A)** or treated with antibodies (Abs) for 1 h **(B)**. Cells were then incubated with FITC-labeled *Enterococcus faecalis* EF-2001 (50 µg/mL) for 2 h, and phagocytic activity was assessed by flow cytometry. Cytochalasin D (CytD, 0.25 µM) was used as a positive control. The phagocytosis index was calculated by multiplying the number of FITC-positive cells by the mean fluorescence intensity (MFI). Data are presented as the mean ± SD (*n* = 3). NC, universal negative control; Isotype control, isotype-matched IgG2 Ab; Anti-Msr1, neutralizing antibody (anti-mouse CD204 Ab). Statistical significance for siRNA-mediated knockdown experiments was determined by one-way ANOVA followed by Dunnett’s multiple comparison test; ***p < 0.001 versus the NC group, whereas comparisons between the isotype control and anti-Msr1 groups were analyzed using an unpaired Student’s *t*-test; ***p < 0.001 versus the isotype control group.

To evaluate the contribution of the candidate receptors to EF-2001 phagocytosis, RAW264 cells with siRNA treatments were incubated with 50 µg/mL FITC-labeled EF-2001 for 2 h. Phagocytic activity was assessed using flow cytometry (**Figure 7A**). The FITC-positive cell counts and MFI are presented separately in **Supplementary Table 1**. Compared with the negative control, the downregulation of *Msr1*, *Cd36*, and *Colec12* significantly decreased phagocytic activity by 72%, 23%, and 20%, respectively (*p* < 0.001). Since *Msr1* knockdown resulted in the strongest suppression of phagocytosis, MSR1 was considered a major receptor for EF-2001 uptake. To confirm this, phagocytic activity was assessed using an MSR1 neutralizing antibody (anti-CD204) (**Figure 7B**). Pre-treatment with the neutralizing or isotype control antibody for 1 h significantly reduced the phagocytic activity by 79% compared with the isotype control group (*p* < 0.001), similar to the siRNA knockdown. Therefore, *Msr1* was identified as the primary receptor for phagocytosis in EF-2001.

### 3.6 Inhibition of intracellular signaling pathways altered cytokine production and phagocytic activity

**Figure 8:**
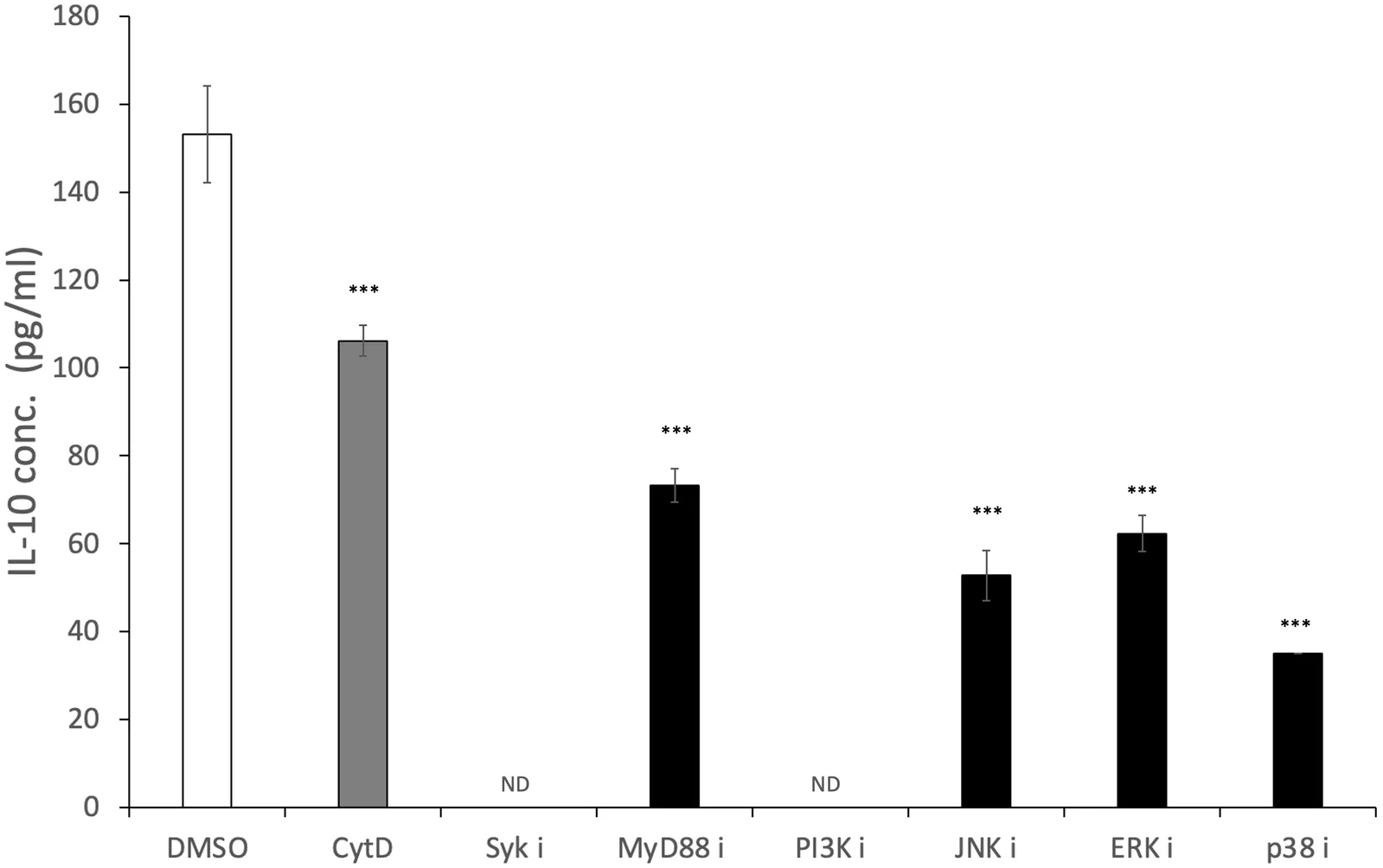
Kinase signaling pathways are involved in *Enterococcus faecalis* EF-2001-induced IL-10 production. RAW264 cells were pre-treated with signal transduction inhibitors for 1 h, followed by stimulation with *Enterococcus faecalis* EF-2001 (50 µg/mL) for 6 h. IL-10 levels in the culture supernatant were measured by ELISA. The following inhibitors were used: piceatannol (Syk inhibitor, 20 µM), TJ-M2010-5 (MyD88 inhibitor, 20 µM), wortmannin (PI3K inhibitor, 0.5 µM), JNK-IN-8 (JNK inhibitor, 10 µM), PD184352 (ERK inhibitor, 10 µM), and VX-745 (p38 inhibitor, 0.5 µM). Cytochalasin D (CytD, 0.25 µM) was used as a positive control. Data are presented as the mean ± SD (*n* = 3). ND, not detected. Statistical significance was determined by one-way ANOVA followed by Dunnett’s multiple comparison test; ***p < 0.001 versus the DMSO group.

**Figure 9:**
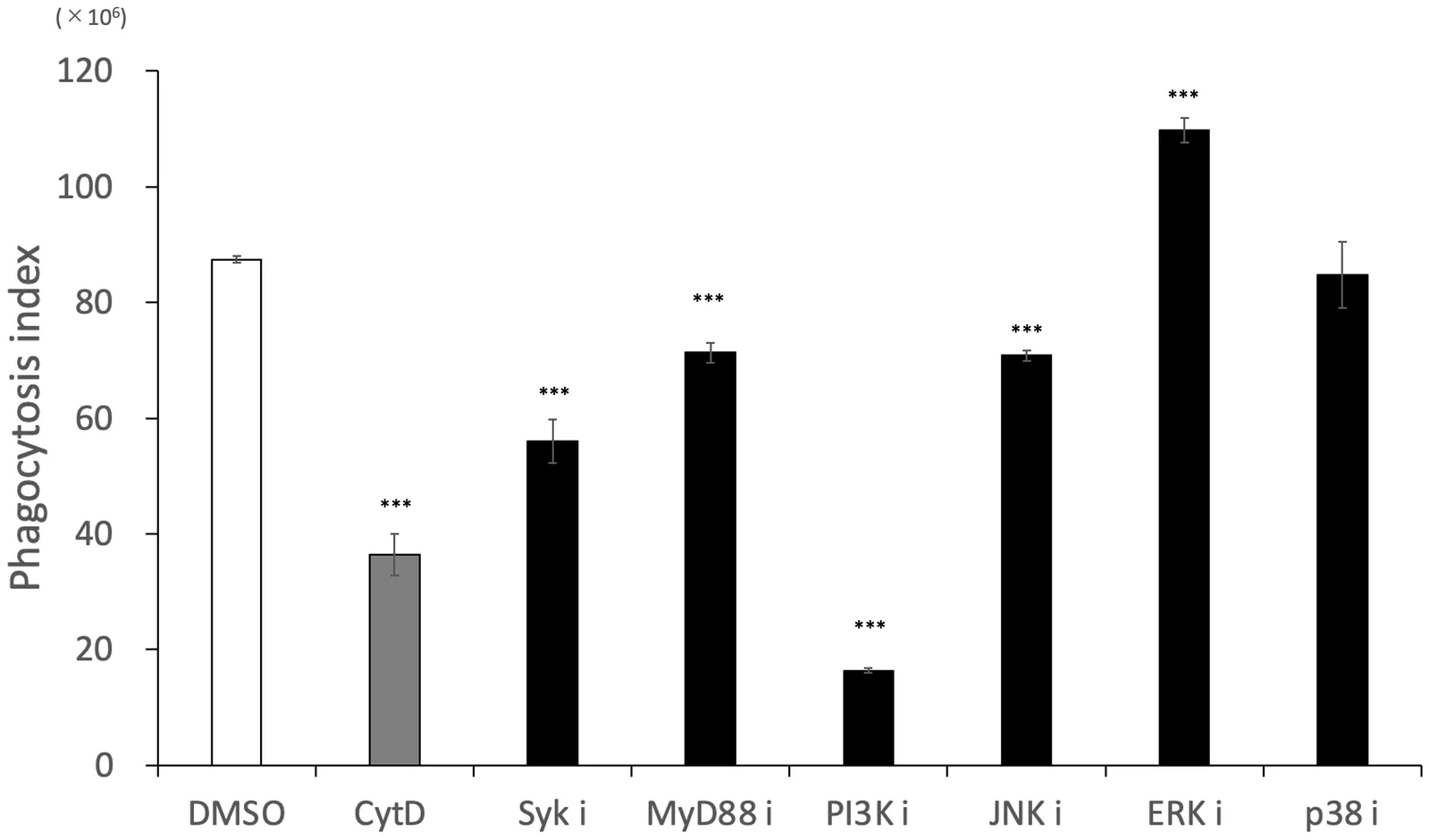
PI3K pathway plays a major role in the phagocytosis of *Enterococcus faecalis* EF-2001. RAW264 cells were pre-treated with signal transduction inhibitors for 1 h, followed by incubation with FITC-labeled *Enterococcus faecalis* EF-2001 (50 µg/mL) for 2 h. Phagocytic activity was assessed by flow cytometry. The following inhibitors were used: piceatannol (Syk inhibitor, 20 µM), TJ-M2010-5 (MyD88 inhibitor, 20 µM), wortmannin (PI3K inhibitor, 0.5 µM), JNK-IN-8 (JNK inhibitor, 10 µM), PD184352 (ERK inhibitor, 10 µM), and VX-745 (p38 inhibitor, 0.5 µM). Cytochalasin D (CytD, 0.25 µM) was used as a positive control. The phagocytosis index was calculated by multiplying the number of FITC-positive cells by the mean fluorescence intensity (MFI). Data are presented as the mean ± SD (*n* = 3). Statistical significance was determined by one-way ANOVA followed by Dunnett’s multiple comparison test; ***p < 0.001 versus the DMSO group.

Among the examined receptor genes, IL-10 secretion was influenced by RNAi-mediated downregulation of candidate receptors. Considering the previously reported anti-inflammatory effects of EF-2001, the downstream signaling pathways involved in the IL-10 response were further investigated. Specific inhibitors of key kinases were selected to inhibit these pathways. RAW264 cells were pre-treated with the specific inhibitors for 1 h, followed by stimulation with 50 µg/mL EF-2001. IL-10 production was significantly reduced by inhibitors targeting MyD88 (52%), JNK (66%), ERK (59%), and p38 (77%) (*p* < 0.001) (**Figure 8**). Syk and PI3K inhibitors substantially reduced IL-10 secretion to below the detection limit (**Figure 8**). Next, the intracellular transduction of phagocytosis was determined. Cells pre-treated with the inhibitors were stimulated with EF-2001, and their phagocytic activities were analyzed by FCM. A different inhibition pattern was observed for IL-10 production. The suppression of signaling pathways significantly reduced phagocytic activity, with reductions of 81% (PI3K), 36% (Syk), 19% (JNK), and 18% (MyD88) (*p* < 0.001) (**Figure 9**). The FITC-positive cell counts and MFI are presented separately in **Supplementary Table 2**. In contrast, p38 inhibition did not affect uptake whereas ERK inhibition significantly but slightly increased uptake. These data suggest that phagocytosis and IL-10 release are involved in intracellular signaling pathways. To determine whether inhibitors and antibody used in this study affect cell viability, cell viability was assessed. No significant differences in cell viability were observed compared to the control group (**Supplementary Figures 2A, B**).

## 4 Discussion

Scavenger receptors on macrophages are involved in the clearance of various ligands and in immune homeostasis via the modulation of inflammatory responses (39, 40). In this study, we explored the corresponding receptors for EF-2001 phagocytosis and evaluated their functions in RAW264 cells to elucidate the underlying mechanisms of immunomodulation. Using bioinformatic analyses, scavenger receptor genes encoding MSR1, CD36, and COLEC12 were found to be candidate receptors for the initial recognition and phagocytosis of EF-2001. Gene silencing of *Msr1* significantly suppressed phagocytosis, whereas silencing of *Cd36* and *Colec12* resulted in slight suppression. Although siRNA incompletely downregulated the gene and protein expression of *Msr1* compared to *Cd36* and *Colec12*, MSR1-mediated phagocytosis was substantially impaired. EF-2001 is predominantly engulfed by macrophages via MSR1. The inhibition of EF-2001 phagocytosis was confirmed using a neutralizing antibody against MSR1. MSR1 was identified as a prominent phagocytic receptor in EF-2001. MSR1 recognizes bacterial components such as LTA or β-glucan (12). These molecules exert immunomodulatory effects. For instance, Curdlan, which is a β-glucan isolated from gram-negative bacteria, induces IL-10 in monocytes although the effects are mediated by other receptors, Dectin-1 or CR3 (41). Some LTAs from Lactobacilli species induce IL-10 as well as TNF-α production with MAPK activations in RAW264.7 cells (42). Considering the similarity of the cytokine profile, LTA of EF-2001 would be a more possible ligand for MSR1. In contrast, heat-inactivated *E. faecalis* strain EC-12 is phagocytosed via the mannose receptor (MR) (43) whereas heat-inactivated *E. faecalis* strain KH2 interacts with CLR, Dectin-2 to elicit wound healing effects (44). MR and Dectin-2 share mannose as a common ligand (45). These results suggest that the recognition of EF-2001 by macrophages may be associated with ligands distinct from other strains within the same *E. faecalis*. To further elucidate the activation mechanisms of EF-2001 and other postbiotics, it is necessary to identify the ligand(s) on EF-2001 responsible for MSR1-mediated recognition.

Among the signaling factors examined, PI3K inhibition had the most profound suppressive effect on EF-2001 phagocytosis. Because PI3K mediates phagocytosis by regulating actin rearrangement, PI3K is critical for phagocytic processes, especially phagosome closure (46). These findings suggest that MSR1 association with EF-2001 transduces its signal to PI3K and induces actin cytoskeleton modulation to internalize EF-2001. In contrast, individual inhibition of the upstream factors of PI3K, MyD88 and Syk led to slight and modest suppression, respectively, in our model. However, in terms of Syk, higher concentrations of piceatannol reduced the phagocytosis of *Cryptococcus neoformans* in mouse BMDM (34). Therefore, the inhibitor has the potential to cause greater abrogation of Syk activity, suggesting the involvement of Syk in EF-2001 phagocytosis. Although piceatannol is 10-fold more specific to Syk than Lyn which is a tyrosine kinase similar to Syk (47), the inhibitor might cause off-target effects. Complementary experiments like knockout/knockdown are needed to confirm the involvement of Syk. Unlike other PRRs, MSR1 lacks a phosphorylation module in intracellular domain (12). It has been reported that complex formation of MSR1 with Fcγ receptors regulates non-opsonic phagocytosis (34). This interaction leads to ITAM phosphorylation within Fcγ receptors and activation of downstream kinase pathways including Syk, PI3K, and MAPKs. In this context, the recognition of EF-2001 by MSR1 may involve Syk-associated signaling, leading to downstream activation. However, the triggering by MSR1 remains a theoretical explanation. Future studies such as Fcγ receptor blockade are needed to clarify the actual interaction. MyD88 is an adaptor molecule for TLR (48) that activates PI3K through its C-terminal binding motif (49). Although the involvement of TLRs in EF-2001 is yet to be investigated, MyD88 appears to contribute slightly to signaling. Alternative pathways independent of MyD88 or Syk may also be involved. In contrast, inhibition of MAPKs did not suppress phagocytosis, although JNK inhibition resulted in limited suppression. Because JNK is involved in cytoskeletal reorganization and actin dynamics (50), it may contribute to phagosome formation through a mechanism that is independent of PI3K-mediated actin control. Unlike previous reports demonstrating the necessity of ERK and p38 for the uptake *C. neoformans* (34), our results showed little or no contribution of MAPKs to EF-2001 phagocytosis. This distinct MAPK requirement may be attributed to the different structures of microbes, such as cell wall components. These data suggested that the MSR1 may be associated with activation of the Syk-PI3K pathway during EF-2001 phagocytosis.

Macrophages regulate the immune response via cytokine production. Peiser et al. reported that production of TNF-α, IL-6 and IL-10 was observed in mouse BMDM following phagocytosis of *Neisseria meningitidis* via MSR1 (51). Given that EF-2001 stimulation exhibited a phenotype similar to that reported previously, our cell line model could reflect immune responses *in vivo*. Here, we focused on the anti-inflammatory responses induced by EF-2001 stimulation. MSR1 knockdown significantly suppressed IL-10 production, suggesting that EF-2001 induced anti-inflammatory responses via MSR1. Our data suggest that the Syk-PI3K pathway may be predominantly involved in the EF-2001-induced anti-inflammatory response. Similarly to the phagocytosis pathway, crosstalk between MSR1 and FcγR could function as a trigger of subsequent signaling steps. Phagocytosis and cytokine induction share overlapping intracellular signaling pathways, including Src family kinases, Syk, PI3K, and MAPK signaling (52), supporting our results. Meanwhile, suppression of IL-10 production was also observed with the moderate inhibition of each MAPK signaling pathway. Specific inhibitors of MAPKs, ERK, JNK, and p38 hamper IL-10 production in macrophages, indicating positive transcriptional regulation of IL-10 (53). A specific inhibitor of MyD88 moderately reduced IL-10 production. Similar to phagocytosis, inhibition of MyD88 implies the presence of other upstream factors such as TLRs. *Cd36* knockdown marginally reduced the phagocytosis of EF-2001, whereas IL-10 production was suppressed. CD36 regulates IL-10 production through intracellular signaling involving the p38 MAPK pathway during the clearance of apoptotic cells without phagocytosis (54). This study implies that CD36-EF-2001 contact is involved in another IL-10 signaling pathway, regardless of EF-2001 phagocytosis. These results suggest that the recognition of EF-2001 leads to an immunomodulatory capacity to induce anti-inflammatory cytokine responses, potentially involving MSR1-mediated Syk-PI3K-MAPKs signaling pathways. However, it is unclear whether the recognition or internalization of EF-2001 is the initial step in transducing signals and producing cytokines, as the current study did not directly investigate the relationship between MSR1 and intracellular signaling pathways. Further studies are required to elucidate these mechanisms.

IL-10 induction by probiotics and postbiotics is associated with beneficial immunomodulatory effects in inflammatory disorders, including intestinal inflammation. *Clostridium butyricum*, a clinically used butyrate-producing probiotic, induces IL-10 production in intestinal macrophages and ameliorates colitis in mice (55). However, administration of live probiotics has occasionally been associated with infectious complications in susceptible hosts (20, 56). In contrast, postbiotics, which consist of non-viable microbial cells or their components, have a lower risk of such complications while retaining their immunomodulatory properties. Postbiotics can improve host immune responses by enhancing IL-10 production (57, 58). EF-2001, a preparation of the heat-inactivated *E. faecalis* strain EF-2001, was also reported to ameliorate intestinal inflammation in experimental models (28, 59). The authors postulated that these effects could be attributed to uncertain anti-inflammatory modulation. Our colleagues discovered that consecutive ingestions of EF-2001 improved reduced gut tight junctions caused by chronic unpredictable mild stresses accompanied by recovery of decreased IL-10 production in mice “We thank Dr. K. Takahashi for providing the latest results (personal communication, March 2026, and under submission).” IL-10 plays a central role in controlling intestinal inflammation, and its deficiency is associated with the onset and exacerbation of inflammatory bowel disease (60). Induction of IL-10 by EF-2001 through MSR1 phagocytosis may explain the molecular mechanism underlying the previously reported therapeutic effects of EF-2001 in ulcerative colitis. Therefore, EF-2001 may be a promising postbiotic for the prevention of inflammatory disorders. Further studies are required to clarify the relationship between the EF-2001-induced cytokine responses and their protective effects. Moreover, we are planning to evaluate the reduction of LPS-induced inflammation by EF-2001 to further dissect the anti-inflammatory mechanism in our future study.

This study has several limitations. First, this study was conducted *in vitro* using RAW264 cells, which are not exactly same as physiological macrophages and may not fully reflect the complex phagocytic mechanisms that occur *in vivo*. Further studies should use primary cultured cells, such as BMDM and peritoneal macrophages isolated from mice, to determine whether similar responses can be reproduced under more realistic conditions. To confirm our findings in human model, macrophages from the human monocyte cell line THP-1 should be used. In addition, it is necessary to differentiate M1 and M2 macrophages from THP-1 in combination with detail cytokine profile analyses to understand more precise mechanisms of inflammatory responses. We are planning to conduct such studies using THP-1 in the future. Second, although we used specific and well-established signaling inhibitors for pathway analysis, it is possible that the inhibitors caused off-target effects even at appropriate concentrations. Future studies should be conducted using complementary approaches, such as genetic knockout using CRISPR or knockdown, to confirm inhibition. Alternatively, immunoblotting should be designed to directly detect the phosphorylation levels of the corresponding molecules after activation with EF-2001. Third, we assumed coordinated activation of multiple receptors such as TLRs and Fcγ receptors but did not verify the contribution of co-receptors to MSR1. With respect to inflammatory cytokines such as TNF-α, it is important to investigate interactions between CD36 and TLR2 or 4 with NF-κB signaling. Further studies are necessary to determine the crosstalk between related molecules by simultaneous repression of them using knockdown combined with blockade antibodies or specific inhibitors in addition to phosphorylation analysis. In terms of statistics, formal tests for normality and homogeneity of variance were not performed because of the small sample size (n=3). Parametric analyses were conducted under the assumptions of approximate normality and homogeneity of variance.

## 5 Conclusion

In the current study, we demonstrated that EF-2001 was recognized and phagocytosed predominantly via MSR1 in RAW264 macrophage-like cells. This process is associated with the induction of IL-10 production through the Syk-, PI3K-, and MAPK-related signaling pathways. These results suggest that EF-2001 could be a favorable postbiotic with an immunomodulatory capacity to suppress inflammatory responses. These findings provide mechanistic insights into how post-biotic components interact with macrophages and contribute to their immune modulation.

## Data Availability

The datasets presented in this study can be found in online repositories. The names of the repository/repositories and accession number(s) can be found in the article/**Supplementary Material**.
