# Correction: Postbiotic *Enterococcus faecalis* EF-2001 induces IL-10 production through macrophage scavenger receptor 1-mediated phagocytosis

**DOI:** 10.3389/fimmu.2026.2013296

**Published:** 2026-10-15

**Authors:** Yuki Kubota, Takumi Tsuboya, Marina Murakami, Shuichiro Uehara, Shigeo Suzuki

**Affiliations:** 1 Department of Pediatric Surgery, Nihon University School of Medicine, Tokyo, Japan; 2 Nihon Berumu Co., Ltd., Tokyo, Japan

**Keywords:** anti-inflammatory cytokine, Enterococcus faecalis EF-2001, immune modulation, lactic acid bacteria, macrophage scavenger receptor 1, phagocytosis, postbiotics, signaling pathway

Author “Yuki Kubota” was erroneously assigned to affiliation “Nihon Berumu Co., Ltd.” This affiliation has now been removed from author “Yuki Kubota”.

The **Conflict of interest** statement was erroneously given as “YK, TT, MM, and SS were employed by Nihon Berumu Co., Ltd.” The correct **Conflict of interest** statement is “TT, MM, and SS were employed by Nihon Berumu Co., Ltd”.

The original version of this article has been updated.

