# Supplementary material for: Postbiotic Enterococcus faecalis EF-2001 induces IL-10 production through macrophage scavenger receptor 1-mediated phagocytosis
Source: Front Immunol. 2026 Aug 20;17:1901007. doi: 10.3389/fimmu.2026.1901007 (PMC13537996; doi:10.3389/fimmu.2026.1901007)
Supplement: Supplementary file 1 [file DataSheet1.pdf]

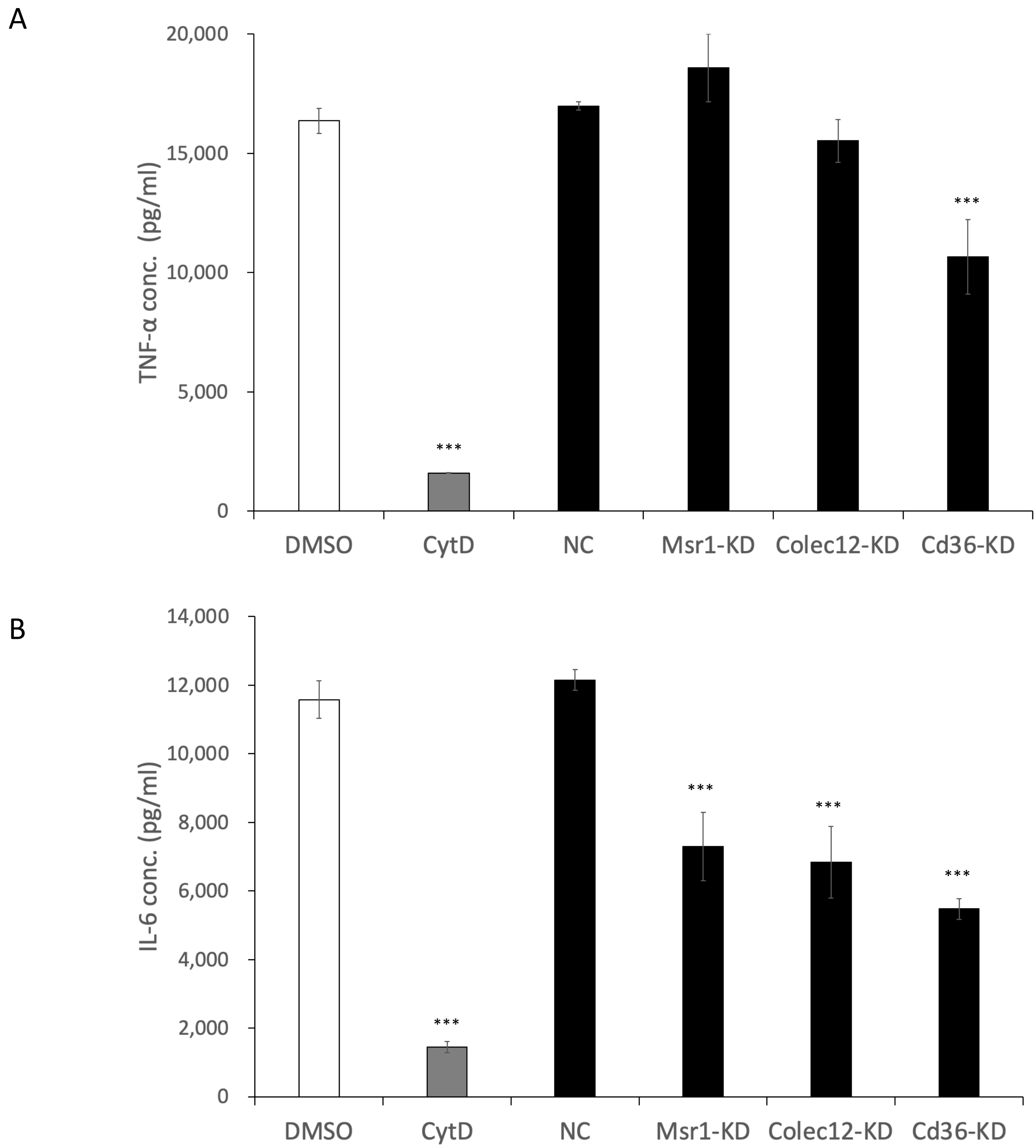

Figure S1 siRNA-mediated silencing of candidate receptors suppresses cytokine production in response to *Enterococcus faecalis* EF-2001.

RAW264 cells were transfected with siRNAs targeting each candidate gene for 24 h, followed by stimulation with *Enterococcus faecalis* EF-2001 (EF-2001) (50 µg/mL). TNF-α (A) and IL-6 (B) levels in the culture supernatant were determined by ELISA at 6 h and 24 h post-stimulation, respectively. 2 µM cytochalasin D (CytD) was used as a positive control. Knockdown of Cd36 significantly and modestly reduced TNF-α ($p < 0.001$) although those of Msr1 and Colec12 did not affect its production (Figure S1A). IL-6 production significantly and moderately declined ($p < 0.001$) by Msr1, Cd36 and Colec12 knockdowns (Figure S1B). Data are presented as the mean ± SD (n=3). NC, universal negative control; ND, not detected. Statistical significance was determined by one-way ANOVA followed by Dunnett's multiple comparison test; ***$p<0.001$ versus the NC group.

A

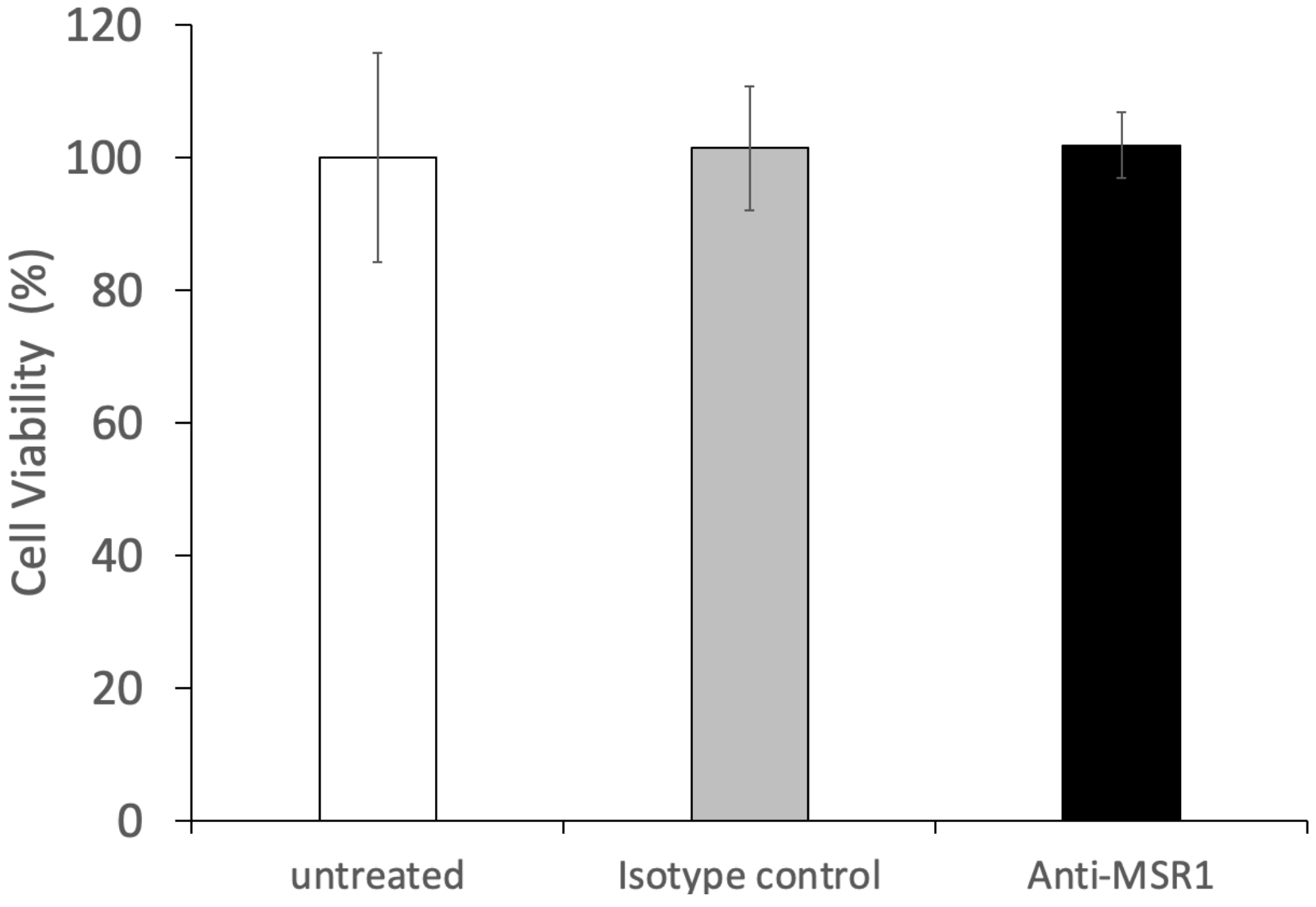

B

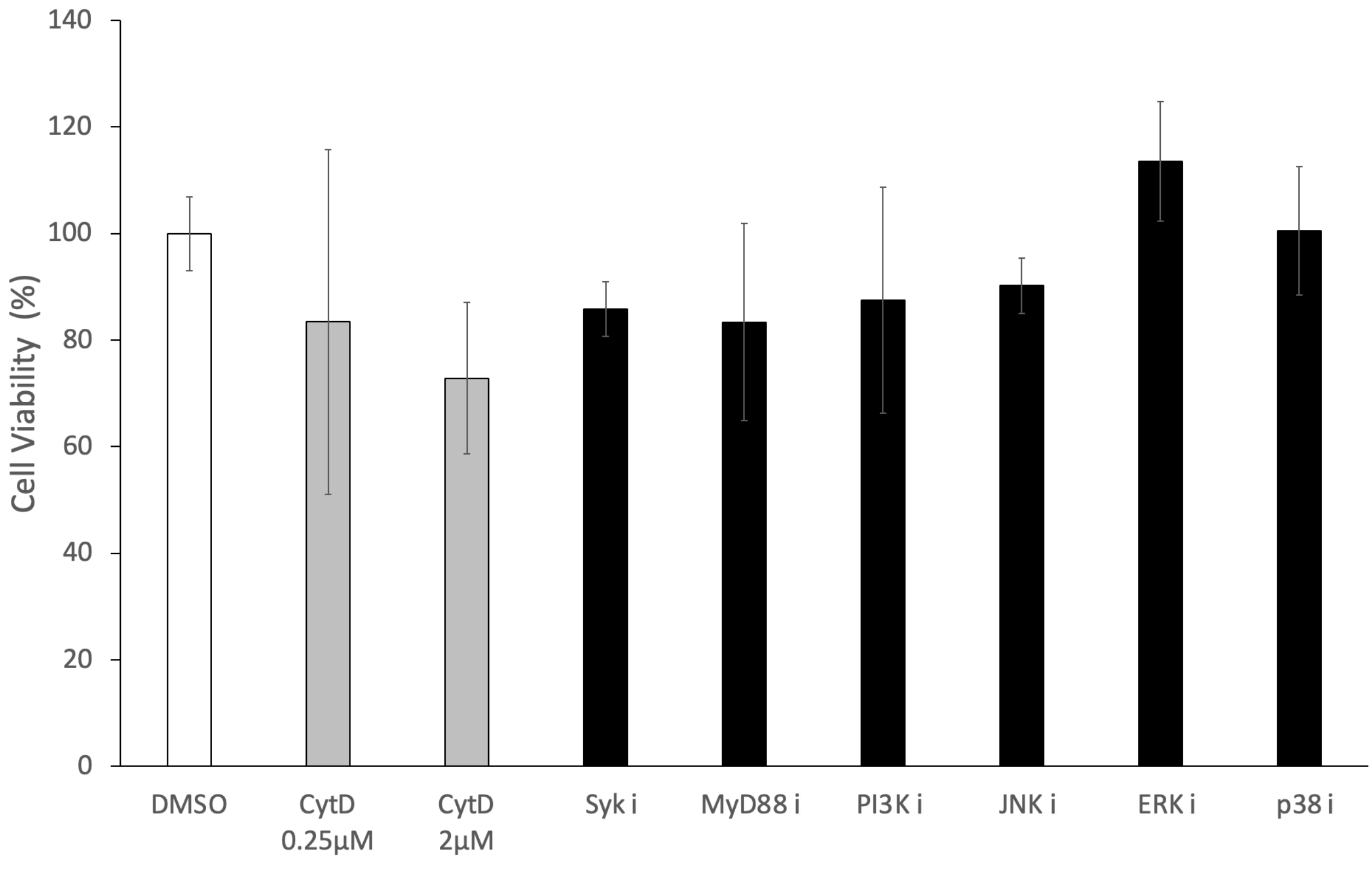

Figure S2 Effects of cytochalasin D, MSR1 neutralizing antibody, and signal transduction inhibitors on RAW264 cell viability.

RAW264 cells were treated with each regent for 1 h, and cell viability was assessed using Cell Counting Kit-8. (A) Cells were treated with anti-MSR1 neutralizing antibody or isotype control antibody (1 μg/mL). Cell viability was calculated relative to untreated cells, which were set to 100%. (B) Cells were treated with cytochalasin D (CytD, 0.25 or 2 μM) or the indicated signal transduction inhibitors. The inhibitors used were piceatannol (Syk inhibitor, 20 μM), TJ-M2010-5 (MyD88 inhibitor, 20 μM), wortmannin (PI3K inhibitor, 0.5 μM), JNK-IN-8 (JNK inhibitor, 10 μM), PD184352 (ERK inhibitor, 10 μM), and VX-745 (p38 inhibitor, 0.5 μM). Cell viability was calculated relative to DMSO-treated cells, which were set to 100%. Data are presented as the mean ± SD (n=3). Statistical significance was determined by one-way ANOVA followed by Dunnett's multiple comparison test; no significant differences were observed compared with the corresponding control group.

Table S1. FITC-positive cell count and mean fluorescence intensity (MFI) for calculating phagocytosis index in siRNA-mediated knockdown and antibody blockade experiments.

	FITC positive cell count	MFI	Phagocytosis Index
**DMSO**	2,218	39,181	86,942,165
**CytD**	1,658	21,370	35,458,479
**NC**	2,309	38,971	89,998,316
**Msr1-KD**	1,415	17,149	24,291,722
**Colec12-KD**	2,151	32,321	69,557,606
**Cd36-KD**	2,099	31,713	66,577,907
**Isotype control**	2,252	38,095	85,794,919
**Anti-MSR1**	1,280	14,491	18,539,780

Table S2. FITC-positive cell count and mean fluorescence intensity (MFI) for calculating phagocytosis index in signal transduction inhibitor experiments.

	FITC positive cell count	MFI	Phagocytosis Index
**DMSO**	2,857	30,604	87,421,098
**CytD**	2,198	16,557	36,458,236
**Syk i**	2,489	22,503	56,056,782
**MyD88 i**	2,723	26,208	71,313,077
**PI3K i**	1,408	11,629	16,369,257
**JNK i**	2,891	24,513	70,861,434
**ERK i**	3,160	34,734	109,745,999
**p38 i**	2,992	28,302	84,742,200
